# Lipid metabolic pathways as lung cancer therapeutic targets: A computational study

**DOI:** 10.3892/ijmm.2011.876

**Published:** 2011-12-30

**Authors:** KOJIRO YANO

**Affiliations:** Faculty of Information Science and Technology, Osaka Institute of Technology, 1-79-1 Kitayama, Hirakata-City, Osaka, Japan

**Keywords:** lung cancer, microarray, lipid metabolism

## Abstract

Inhibitors of lipid metabolic pathways, particularly drugs targeting the mevalonate pathway, have been suggested to be valuable in enhancing the effectiveness of epidermal growth factor receptor-tyrosine kinase inhibitors (EGFR-TKIs) and these compounds may also be effective in patients with inherent or acquired resistance to EGFR-TKIs. The present study examined gene expression profiles in lung adenocarcinoma to characterize the interaction between growth factor signals and lipid metabolic pathways at the transcriptional level. Gene expression correlation analysis showed that genes involved in the mevalonate pathway and unsaturated fatty acid synthesis were negatively correlated with the expression of EGFR, MET and other growth factor receptor genes, as well as with the expression of genes involved in cell migration and adhesion. On the other hand, the expression of genes related to cell cycle progression, DNA repair and DNA replication were positively correlated with the metabolic pathway genes mentioned above, and a significant number of such genes had promoter domains for nuclear factor Y (NFY). Genes whose expression showed a positive correlation with NFY expression and mevalonate pathway genes were found to exhibit protein-protein interactions with several ‘hub’ genes, including BRCA1, that have been associated with both lung cancer and cell division. These results support the idea that inhibition of lipid metabolic pathways may be valuable as an alternative therapeutic option for the treatment of lung adenocarcinoma, and suggest that NFY is a possible molecular target for such efforts.

## Introduction

Lung adenocarcinoma accounts for about half of all non-small cell lung cancer (NSCLC) cases and is one of the major causes of death in developed countries (1). Epidermal growth factor receptor (EGFR) tyrosine kinase inhibitors (TKIs) have been intensively assessed over the past several years as targeted agents for advanced NSCLC. Whereas EGFR-TKIs are highly effective in the treatment of adenocarcinoma associated with specific EGFR mutations that cause sustained receptor activity, drug effectiveness is significantly lower in patients without the activating mutations, and even patients with the mutations frequently develop resistance to EGFR-TKI (2). Therefore, new therapeutic targets that can overcome inherent or acquired resistance to EGFR-TKIs are highly desirable. Recently, it has been suggested that acquired resistance to EGFR-TKIs may be related to amplification of a hepatocyte growth factor (HGF) receptor, termed MET (3). HGF expression can induce EGFR-TKI resistance to lung adenocarcinoma cells with EGFR-activating mutations (4), and MET inhibition can reduce proliferation of lung adenocarcinoma cell lines that show resistance to EGFR-TKIs (3). MET amplification occurs in about 20% of NSCLC patients and is associated with poor survival.

The lipid metabolism pathway may also modulate the effectiveness of EGFR-TKIs in lung adenocarcinoma patients. It has been suggested that lipid-lowering drug statins may reduce cancer risk (5), and a large case-control study of US veterans found that this may be true for lung cancer (6), although some reports claim otherwise (7,8). *In vitro* studies have shown that inhibition of the mevalonate pathway by statins reduces EGFR autophosphorylation (9), downstream AKT signaling (10), and EGF-induced RhoA translocation to the plasma membrane (11). Enhancement of EGFR-TKI effectiveness by statins seems to occur not only in cells with EGFR-activating mutations but also in EGFR-TKI-resistant NSCLC cell lines (12). The mechanism of EGFR signaling inhibition is not fully characterized, but reduced prenylation of small GTP-binding proteins may be of importance (13). However, depletion of cholesterol in the plasma membrane is known to increase EGFR signaling activity, perhaps by releasing EGFR from lipid rafts and inhibiting receptor internalization (14,15). This suggests that the lipid metabolism pathway can influence EGFR signaling in both a positive and negative manner.

This study sought to characterize the lipid metabolism pathway in lung adenocarcinoma using gene expression correlation analysis of microarray data. More specifically, pathway genes that show associations with EGFR or MET were examined in detail, because EGFR and MET are among the best-studied growth signals in lung cancer patients. Gene expression profiles have been used to classify lung cancer (16), to discover gene sets which are predictive of disease prognosis (17), and to investigate molecular mechanisms of disease progression (18). However, large-scale analysis of the association between metabolic and growth factor signaling pathways has not been conducted in lung cancer tissue. In the present study, a set of lipid metabolism pathway genes, the expression of which are highly correlated with EGFR or MET, were first selected. Next, genes in the microarray dataset showing significant correlation with selected genes were examined in terms of functional properties. Finally, possible regulatory mechanisms of correlated expression were inferred using known transcription factor target sequences. This type of analysis predicts how the lipid metabolic pathway may functionally interact with EGFR, MET, and other biological processes in lung cancer cells, and offers an insight into the roles of EGFR and MET inhibition in lung cancer therapeutics.

## Materials and methods

### Microarray data

The microarray dataset GSE10072 (19) from the Gene Expression Omnibus (20) was used for analysis. The dataset contains expression profiles of 58 tumor and 49 non-tumor tissues. The information was originally obtained using the Affymetrix Human Genome U133A Array. The data from 22,215 probes in the array were normalized using the quantile normalization function (quantilenorm) of the Matlab Bioinformatics Toolbox (MathWorks, Natick, MA).

### Classification of genes by Gene Ontology

The DAVID functional annotation tool [version 6.7b (21,22)] was used to classify gene sets by Gene Ontology identifiers or using UCSC transcription factor binding sites (23). Functional categories with a Benjamini-Hochberg statistic (24) of <0.025 were considered statistically significant.

### Statistical analysis

Pearson correlation coefficients were calculated using the ‘corr’ function from Matlab. The 2.5th and 97.5th percentiles of coefficients for 100,000 pairwise combinations between randomly selected genes in the dataset were −0.379 and 0.428, respectively, and these were used as threshold values for significantly negative and positive correlations. Two-sample t-testing was achieved using the ‘ttest2’ function from Matlab.

## Results

### Correlation of lipid metabolism genes with EGFR expression

**Figure 1:**
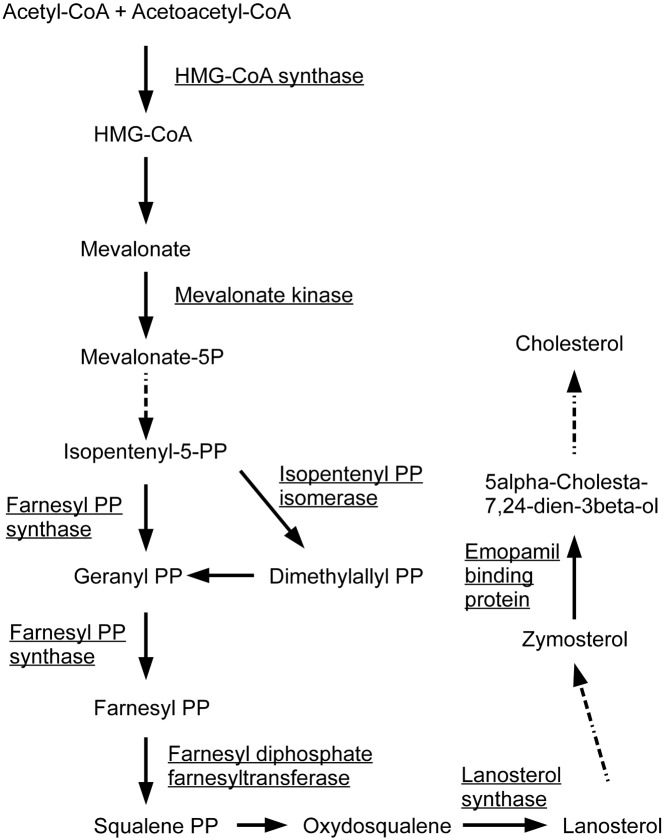
The mevalonate pathway. Enzymes, the encoding genes of which were negatively correlated with EGFR or MET expression are underlined. Broken arrows indicate that more than one reaction is involved. P and PP indicate phosphate and pyrophosphate, respectively.

**Figure 2:**
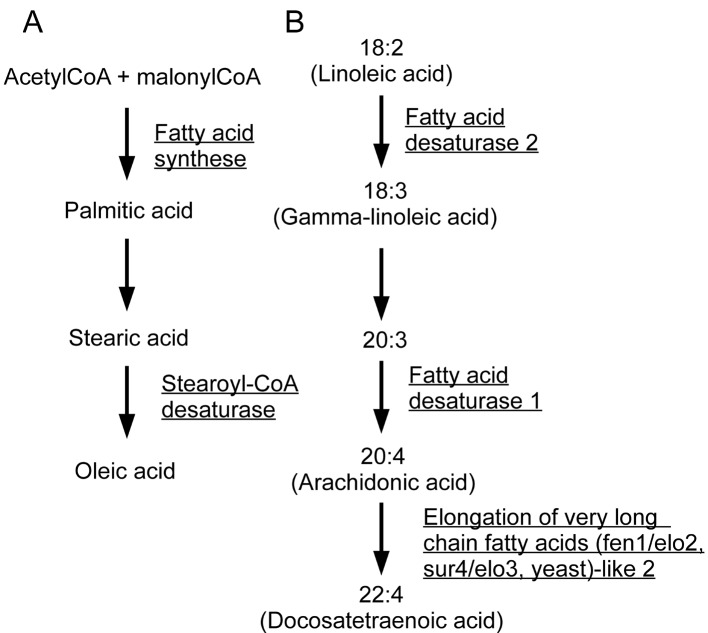
Fatty acid synthesis pathways. Enzymes, the encoding genes of which were negatively correlated with EGFR or MET expression are underlined. (A) Synthesis of a monounsaturated fatty acid, using oleic acid as an example. (B) Synthesis of a polyunsaturated fatty acid, using docosatetraenoic acid as an example.

A total of 301 genes classified as ‘lipid metabolic process’ (GO:0006629) by gene ontology were selected and Pearson correlation coefficients were calculated between the expression of such genes and EGFR and MET. Although no gene showed a positive correlation with EGFR or MET expression, eight and nine such genes displayed a negative correlation with EGFR and MET expression, respectively, in cancer samples (Table I). The negative correlations were not evident in normal lung samples, except for MVK, which showed a significant negative correlation with MET in both cancerous and normal cells. Among the negatively correlated genes, HMG-coenzyme A synthase 1 (HMGCS1), farnesyl-diphosphate farnesyltransferase 1 (FDFT1), farnesyl diphosphate synthase (FDPS), isopentenyl-diphosphate δ isomerase 1 (IDI1), lanosterol synthase (LSS), emopamil binding protein (EBP) and mevalonate kinase (MVK) are known to be involved in the first steps of steroid biosynthesis (Fig. 1). FAS and stearoyl-CoA desaturase (SCD) mediate the synthesis of monounsaturated fatty acids from acetyl-CoA, and fatty acid desaturase 1 (FADS1), fatty acid desaturase 2 (FADS2), and elongation of very long chain fatty acids (fen1/elo2, sur4/elo3, yeast)-like 2 (ELOVL2) catalyze the production of polyunsaturated fatty acids, including arachidonic acid (Fig. 2). Fatty acid 2-hydroxylase (FA2H) is involved in sphingolipid metabolism and mutations in this gene are known to cause leukodystrophy, whereas phosphatidylglycerophosphate synthase 1 (PGS1) is involved in glycerophospholipid metabolism, synthesizing phosphatidyl-glycerophosphate from CDP-diacylglycerol. These results suggest that EGFR and MET are closely, but negatively, associated with the expression of a variety of fatty acid biosynthesis genes in lung adenocarcinoma tissue.

### Functional gene categories associated with lipid metabolism genes anti-correlated to EGFR

Next, associations of the ‘anti-EGFR/MET’ lipid metabolism genes with other genes were evaluated by calculation of the intergene Pearson correlation coefficients in lung cancer samples. Table II shows the number of genes demonstrating significant positive or negative associations with mevalonate pathway genes (FDFT1, FDPS, HMGCS1, IDI1, LSS, EBP and MVK).

Among these seven genes, FDPS, HMGCS1, IDI1 and MVK, all of which mediate farnesyl pyrophosphate synthesis from mevalonate, showed particularly large numbers of correlated genes. In addition, 166 genes in the microarray dataset displayed significant positive associations with three or more of the mevalonate pathway genes. According to DAVID, gene functional categories were dominated by GO Biological Processes related to the cell cycle, DNA replication, response to DNA damage, and lipid metabolism, suggesting close links between the regulation of cell division and cholesterol biosynthesis (Table III). On the other hand, 235 genes had significant negative associations with three or more of the mevalonate pathway genes. The functional categories were principally related to cell adhesion, cell migration, blood vessel development, extracellular matrix synthesis, and defense responses (Table III). This gene set also included regulators of cell proliferation, including endothelin receptor type A (EDNRA), platelet-derived growth factor receptor, α polypeptide (PDGFRA), protein kinase Cα (PRKCA), ras-related C3 botulinum toxin substrate 2 (RAC2), transforming growth factor β, receptor II (TGFBR2), and vitamin D receptor (VDR). These data may suggest that mevalonate pathway genes were negatively associated with processes mediating signal transduction from the extracellular space, but positively associated with pathways involving the nucleus. Similarly, anti-EGFR/MET lipid metabolism genes involved in fatty acid synthesis (FADS1, FADS2, FASN, SCD, ELOVL2, PGS1 and FA2H) were evaluated (Table II). Most of these genes showed smaller numbers of correlations than genes of the mevalonate pathway. Only 18 and 35 genes displayed significant positive and negative correlations, respectively, with three or more of the fatty acid synthesis genes. The positively correlated genes belonged to sets of functional categories similar to those positively correlated with mevalonate pathway genes (Table III); these were genes of the cell cycle, cell division and lipid metabolism. No functional category was significantly enriched in negatively correlated genes.

### Transcriptional regulatory mechanisms associated with anti-EGFR lipid metabolism genes

Gene expression correlation analysis showed that lipid metabolism genes were associated with specific biological processes, particularly the cell cycle. To determine a possible mechanism of correlated expression, enrichment of predicted transcription factor binding sites was examined by DAVID. It was found that genes positively associated with mevalonate pathway genes were enriched in the NFY binding site, with a Benjamini score of 3.4E-8. To examine the relationship between NFY and genes positively correlated with mevalonate pathway genes, a search was instituted for genes showing significant positive correlations with NFY. As NFY is composed of subunits encoded by three genes, NFYA, NFYB and NFYC, genes with positive correlations with at least one subunit were selected. Respectively 202, 889 and 133 genes were found to display a correlation with NFYA, NFYB and NFYC, and, in total, 1,166 genes displayed significant positive correlations with one or more of the NYF subunit genes. For each gene identified, Pearson correlation coefficients were calculated with respect to genes positively correlated with mevalonate pathway genes, and the number of significant positive correlations was enumerated. This disclosed that 53 genes showed positive correlations with 81 or more of mevalonate pathway-associated genes. This threshold of 81 is the top 2.5th percentile of the number of mevalonate pathway genes positively correlated with each gene in the microarray dataset. These 53 genes will be simply termed ‘NFY-correlated genes’ below.

**Figure 3:**
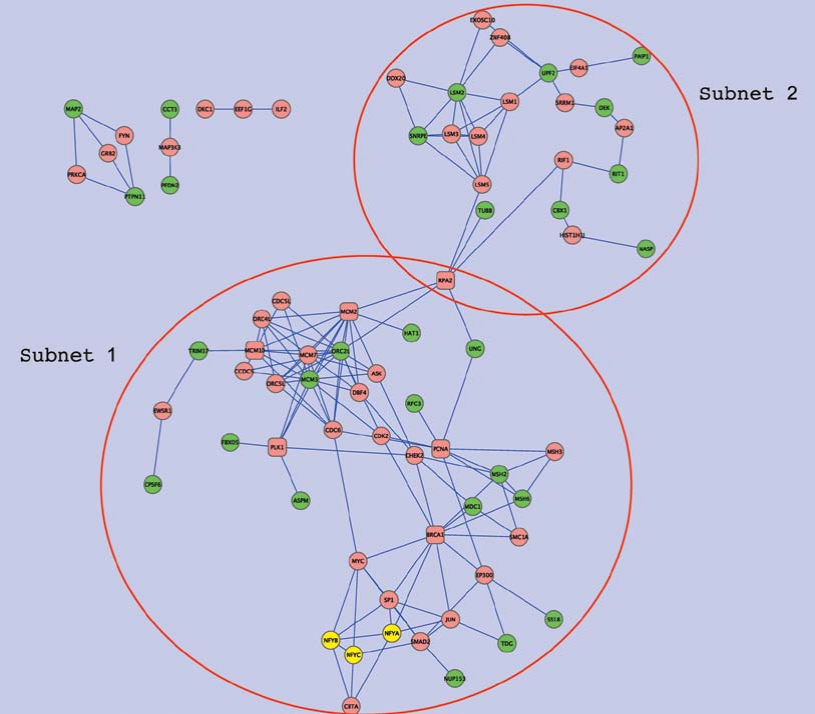
Protein-protein interaction network of NFY and associated genes. Yellow, green and pink nodes are NFY genes, genes correlated with NFY and mevalonate pathway genes, and genes with known protein-protein interactions with NFY genes or NFY-correlated genes. Red round rectangles are ‘hub’ genes (please see the main text). The red circles are subnets 1 and 2; these are shown on a larger scale in Figures 4 and 5, respectively.

**Figure 4:**
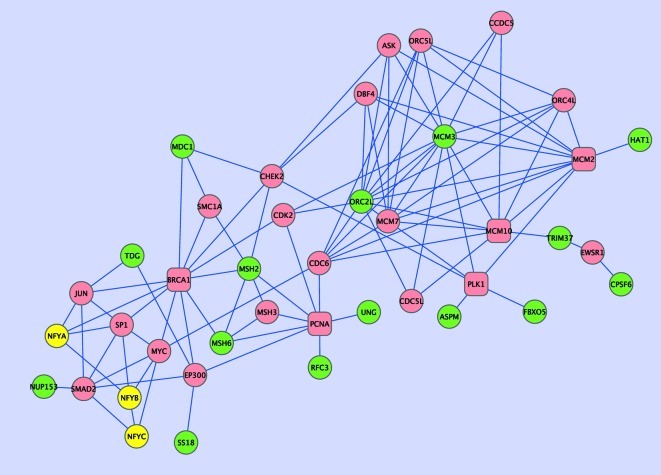
Subnet 1 of the protein-protein interaction network of NFY and associated genes. Notations for nodes are as in Figure 3.

**Figure 5:**
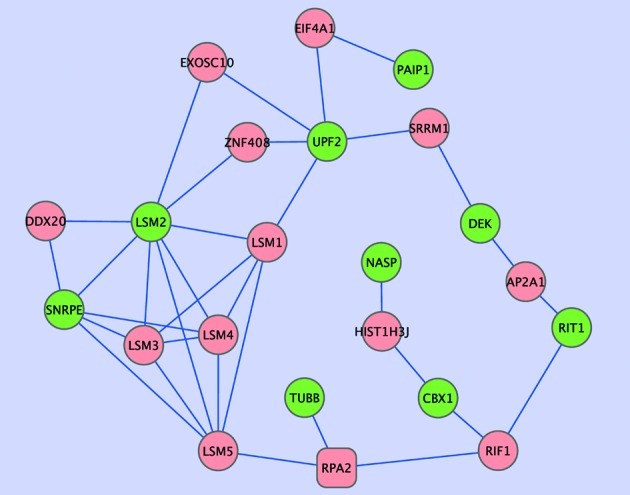
Subnet 2 of the protein-protein interaction network of NFY and associated genes. Notations for nodes are as in Figure 3.

A literature search found no reported direct physical association between NFY and any of the 53 gene products. However, according to DAVID, many of these genes were related to DNA metabolic processes, DNA repair, or mRNA metabolism (Table IV). To account for the observed associations between NFY and NFY-correlated genes, known protein interactions were sought using Genes2Networks (25). Fig. 3 shows the overall network, formed by NFY genes, NFY-correlated genes, and intermediate genes which connect these two gene sets. Extracts from the network, subnets 1 and 2, are shown in Figs. 4 and 5, respectively. Subnet 1 has 15 NFY-correlated genes showing relatively close associations with NFY genes in the interaction network (Table V). Six such genes are involved in DNA repair and five are associated with either the cell cycle (ASPM, FBXO5), DNA metabolic processes (ORC2L, HAT1), or both (MCM3). In this subnetwork, several intermediate or ‘hub’ genes were closely connected to the NFY-correlated genes. Namely, PCNA and BRCA1 were connected to four of the NFY-correlated genes, and each of MCM10, PLK1, MCM2 and RPA2 to three. In addition to these hub genes, CHEK2, CDK2, MCM7, CDC6, EP300 and ORC4L were connected to two of the NFY-correlated genes as well as to two hub genes. Of these genes, PCNA, MCM2, CDK2 and MCM7 showed significantly negative correlations with EGFR (Pearson coefficients, −0.446, −0.399, −0.381 and −0.401, respectively), whereas PLK1, MCM2 and CDK2 displayed significantly negative correlations with MET (Pearson coefficients, −0.373, −0.486 and −0.495, respectively). Moreover, the mean Pearson coefficients of all hub genes were −0.252 for EGFR and −0.240 for MET, both of which were significantly lower than the means for all genes in the dataset (−0.0089 for EGFR and −0.0313 for MET; P=1.678E-4 and 0.0029 by t-tests, respectively), demonstrating negative associations between hub genes and growth signals. Subnet 2 includes nine of the NFY-associated genes that were only distantly connected with NFY genes in the protein-protein interaction network. Five of these genes were related to RNA metabolic processes (PAIP1, SNRPE, DEK, UPF and LSM2) and two genes encoded proteins with histone-binding properties (NASP and CBX1). In this subnetwork, LSM1 showed high connectivity, displaying two edges with the NFY-correlated genes, and three with other intermediate genes. LSM1 is highly expressed in lung cancer and mesothelioma, and LSM1 inhibition retards tumor growth (26). Four other LMS genes were present in the subnet but there was no evidence of association with lung cancer.

## Discussion

In the present study, gene expression correlation patterns predicted that mevalonate metabolism and fatty acid synthesis processes were negatively associated with expression of EGFR and MET, but positively associated with cell division. Promoter analysis suggested that the NFY transcription factor may be involved in the regulation of genes involved in mevalonate metabolism, and the processes positively associated with them. Finally, gene expression correlation patterns and protein-protein interaction data indicate that the transcriptional regulation by NFY may be mediated by its interactions with other regulators of DNA metabolic processes and cell cycle genes.

The negative correlations between growth factor signaling and lipid metabolic pathways reported here seem to indicate an inhibitory effect of cholesterol on EGFR pathways in lung adenocarcinoma. Polyunsaturated fatty acids, such as oleic acid, are also known to inhibit the EGFR pathway, although the effects depend both on particular combinations of fatty acids and the cell type (27–29). In lung adenocarcinoma, the mevalonate pathway synthesizes more non-sterol and fewer sterol products than seen in fibroblasts (30). This can result in a higher degree of prenylation of small GTP-binding proteins, and reduced levels of plasma membrane cholesterol, possibly leading to enhanced EGFR activity. Mevalonate metabolites can also influence the expression of metabolic genes through the intermediacy of the liver X receptor (LXR). For example, LXR can activate FDPS synthesis (31), but LXR is inhibited by geranylgeraniol (32), which is produced from isopentenyl-PP and farnesyl-PP. Indeed, expression of NR1H3 (LXR-α) showed a significant correlation with FDPS and EBP synthesis in lung cancer samples but not in normal lung samples (data not shown), suggesting a cancer-specific regulation of mevalonate pathway genes by LXR-α.

The positive correlations seen between the lipid metabolic pathway and cell division-related processes appear to be consistent with previous experimental evidence. Pravastatin is known to inhibit DNA synthesis, whereas addition of geranylgeranylpyrophosphate restores such synthesis and promotes the G1/S transition (33). However, inhibition of farnesyl-protein transferase induces p21 expression and G1 blockade in a p53-dependent manner, suggesting that regulation of the cell cycle by mevalonate metabolites occurs at both the transcriptional and translational levels. In lung carcinoma cell lines, farnesyl transferase inhibitors block farnesylation of centromeric proteins and inhibit the association of such proteins with microtubules (34). In retinoblastoma gene-deficient thyroid tumors, FDPS is overexpressed, leading to increased isoprenylation and activation of N-Ras and induction of the DNA damage response (35). These experimental findings seem to suggest that mevalonate metabolites can directly regulate the expression of genes related to cell division as well. Unsaturated fatty acids are also known to increase cell proliferation (36) (37), although the mechanism of such action is not clear. One possibility is that increased activity of intracellular signaling cascades, such as those mediated by intracellular calcium (38) or AKT (39), may enhance the response of cells to mitogenic signals. However, unsaturated fatty acids are substrates for lipid peroxidation and may cause DNA damage in lung cancer cells (40–42). This may lead, in turn, to apparent (thus not real) correlated expression of unsaturated fatty acid metabolism genes and DNA damage response genes.

Transcription factor binding sequence analysis suggested that NFY may have a considerable influence on associations of lipid metabolism genes. NFY is a ubiquitous transcriptional factor which recognizes promoter CCAAT boxes (43). NFY is known to be involved in transcriptional regulation of a wide range of genes, but the regulatory roles of NFY in lipogenesis, the cell cycle, DNA repair, and DNA synthesis are of particular interest in the present context. In lipogenic gene regulation, NFY often functions with SREBPs and SP1 (44), and recent genome-wide scanning of SREBP1, SP1 and NFY occupancy showed that NFY shares about 20 and 40% of target genes with SREBP1 and SP1, respectively, in HepG2 cells (45). In the lung adenocarcinoma dataset, some mevalonate pathway genes displayed significant correlation with SREBP1 and SREBP2, but not SP1 (data not shown), suggesting possible coordinated regulation of such genes by NFY and SREBPs in cancer cells.

The regulation of cell cycle and DNA metabolism genes by NFY is also well documented. Expression of a dominant-negative NFY subunit significantly decreased the number of cells entering the S-phase and delayed the progress of this phase, resulting in retarded cell growth (46). NFY seems be involved in induction of S-phase-specific transcription, such as that resulting in synthesis of ribonucleotide reductase R2 (47), histone H3 (48), and cyclin B1 (49). NFY also mediates genotoxic stress-induced gene expression in a p53-independent manner (50), and suppresses gene expression in the presence of active p53 (51), suggesting a functional dependency on co-regulators. Therefore, it was important to define proteins interacting with NFY in the lung cancer cells of the present study. Combined analysis of gene expression correlation and protein-protein interaction identified several ‘hub’ genes which displayed high connectivity with NFY-correlated genes and other hub genes. Importantly, many of the hub genes have been associated with lung cancer. These include BRCA1 (52,53), PCNA (54,55), PLK1 (56,57), MCM2 (58), CHEK2 (59,60), CDK2 (61) and MCM7 (62), suggesting that the network discovered here is likely to be involved in progression of lung cancer. As some such genes were also sensitive to inhibition of the mevalonate pathway [BRCA1 (63), PCNA (64), MCM2 (65), CDK2 (66) and MCM7 (67)], hub genes may also be involved in the antitumor effects of pathway inhibitors in lung cancer. These hub genes do not have direct links to NFY-correlated genes and, although functional association with NFY has been experimentally shown for BRCA1 (68), CDK2 (49,69) and EP300 (70), other hub genes likely interact with NFY through intermediate genes, the expression of which was found to be correlated with that of NFY.

Finally, the results presented in this article have several important clinical implications for the treatment of lung adenocarcinoma. First, the data support the importance of lipid metabolic pathway inhibition in adenocarcinoma patients, particularly in those insensitive to anti-EGFR therapy or patients who have developed resistance to such therapy. The effects of chemotherapy may be enhanced by downregulating genes related to cell division. Some of the hub genes identified in this article are already known as lung cancer markers, but exploration of the activity of combinations of such genes should better indicate the parts of the network that are active or inactive in cancer cells, thus possibly increasing therapeutic predictive power. Finally, drugs targeting NFY may be useful to improve the efficacy of other chemotherapeutic agents, by blocking multiple pathways related to lung carcinogenesis. The roles played by NFY in a variety of cancers have been highlighted in recent reports (71,72), and I believe that a new therapeutic strategy based on inhibition of NFY warrants further research and development.

## Figures and Tables

**Table I:** Lipid metabolic process genes negatively correlated with EGFR or MET.

Symbol	Name	Cancer^a^	Normal^a^
EGFR			
FA2H	Fatty acid 2-hydroxylase	−0.442	−0.073
FADS2	Fatty acid desaturase 2	−0.419	0.181
FDFT1	Farnesyl-diphosphate farnesyltransferase 1	−0.414	−0.093
FDPS	Farnesyl diphosphate synthase	−0.402	−0.321
HMGCS1	HMG-coenzyme A synthase 1	−0.559	−0.224
IDI1	Isopentenyl-diphosphate δ isomerase 1	−0.438	−0.084
LSS	Lanosterol synthase	−0.402	−0.115
SCD	Stearoyl-CoA desaturase	−0.399	−0.021
MET			
EBP	Emopamil binding protein	−0.441	0.245
ELOVL2	Elongation of very long chain fatty acids (fen1/elo2, sur4/elo3, yeast)-like 2	−0.427	−0.146
FADS1	Fatty acid desaturase 1	−0.411	0.115
FADS2	Fatty acid desaturase 2	−0.596	−0.099
FASN	Fatty acid synthase	−0.526	0.108
IDI1	Isopentenyl-diphosphate δ isomerase 1	−0.463	0.301
LSS	Lanosterol synthase	−0.390	0.117
MVK	Mevalonate kinase	−0.380	−0.391
PGS1	Phosphatidylglycerophosphate synthase 1	−0.421	−0.061

a Values in the columns show the Pearson correlation coefficients for the respective growth factor genes.

**Table II:** Number of genes significantly correlated with lipid metabolism genes.

Symbol	Positive	Negative
Mevalonate pathway genes		
FDFT1	68	162
FDPS	438	480
HMGCS1	372	381
IDI1	505	573
LSS	166	192
EBP	294	431
MVK	3012	1462
Fatty acid synthesis pathways genes		
ELOVL2	1428	459
FA2H	87	93
FADS1	182	214
FADS2	130	200
FASN	67	325
PGS1	147	127
SCD	152	188

**Table III:** Enriched GO biological process terms showing significant correlations with mevalonate and fatty acid synthesis pathway genes.

Term	Count	Benjamini
Enriched GO biological process terms in genes positively correlated with the mevalonate pathway genes		
GO:0007049, cell cycle	67	3.56E-37
GO:0000278, mitotic cell cycle	46	4.44E-36
GO:0022403, cell cycle phase	45	1.01E-32
GO:0007067, mitosis	38	1.21E-31
GO:0000279, M phase	41	1.34E-31
GO:0000087, M phase of mitotic cell cycle	38	1.42E-31
GO:0022402, cell cycle process	55	2.26E-29
GO:0051301, cell division	37	3.18E-29
GO:0006259, DNA metabolic process	43	1.37E-15
GO:0006260, DNA replication	24	2.92E-13
GO:0000075, cell cycle checkpoint	15	5.30E-13
GO:0007051, spindle organization and biogenesis	11	5.83E-13
GO:0007017, microtubule-based process	22	2.24E-12
GO:0000074, regulation of progression through cell cycle	31	2.71E-12
GO:0051726, regulation of cell cycle	31	2.92E-12
GO:0006996, organelle organization and biogenesis	44	2.00E-11
GO:0000070, mitotic sister chromatid segregation	11	8.75E-11
GO:0000819, sister chromatid segregation	11	1.19E-10
GO:0007059, chromosome segregation	13	3.18E-10
GO:0006974, response to DNA damage stimulus	23	3.46E-10
GO:0000226, microtubule cytoskeleton organization and biogenesis	14	4.49E-10
GO:0007088, regulation of mitosis	13	1.03E-09
GO:0051276, chromosome organization and biogenesis	24	2.02E-09
GO:0009719, response to endogenous stimulus	23	2.09E-08
GO:0007093, mitotic cell cycle checkpoint	9	2.01E-07
GO:0016126, sterol biosynthetic process	9	2.50E-07
GO:0007010, cytoskeleton organization and biogenesis	24	5.16E-07
GO:0051325, interphase	12	7.14E-07
GO:0006281, DNA repair	17	2.01E-06
GO:0006261, DNA-dependent DNA replication	12	2.29E-06
GO:0016125, sterol metabolic process	11	2.68E-06
GO:0051329, interphase of mitotic cell cycle	11	4.48E-06
GO:0007052, mitotic spindle organization and biogenesis	6	1.13E-05
GO:0006694, steroid biosynthetic process	10	2.43E-05
GO:0006695, cholesterol biosynthetic process	7	2.70E-05
GO:0006270, DNA replication initiation	7	8.05E-05
GO:0009987, cellular process	150	1.10E-04
GO:0008203, cholesterol metabolic process	9	1.25E-04
GO:0044237, cellular metabolic process	112	3.23E-04
GO:0016043, cellular component organization and biogenesis	52	3.59E-04
GO:0006139, nucleobase, nucleoside, nucleotide and nucleic acid metabolic process	68	3.63E-04
GO:0008202, steroid metabolic process	12	3.69E-04
GO:0044238, primary metabolic process	111	7.69E-04
GO:0048015, phosphoinositide-mediated signaling	9	1.33E-03
GO:0006268, DNA unwinding during replication	5	1.42E-03
GO:0031570, DNA integrity checkpoint	6	1.75E-03
GO:0007018, microtubule-based movement	9	1.87E-03
GO:0000910, cytokinesis	6	1.96E-03
GO:0032508, DNA duplex unwinding	5	2.19E-03
GO:0032392, DNA geometric change	5	2.19E-03
GO:0008610, lipid biosynthetic process	13	2.45E-03
GO:0008152, metabolic process	117	3.33E-03
GO:0008299, isoprenoid biosynthetic process	5	3.38E-03
GO:0030705, cytoskeleton-dependent intracellular transport	9	5.04E-03
GO:0031577, spindle checkpoint	4	5.08E-03
GO:0006066, alcohol metabolic process	13	9.20E-03
GO:0042770, DNA damage response, signal transduction	6	1.05E-02
GO:0043283, biopolymer metabolic process	77	1.33E-02
GO:0000077, DNA damage checkpoint	5	1.65E-02
GO:0006950, response to stress	25	1.67E-02
GO:0006720, isoprenoid metabolic process	5	1.85E-02
Enriched GO biological process terms in genes negatively correlated with the mevalonate pathway genes		
GO:0022610, biological adhesion	61	1.51E-25
GO:0007155, cell adhesion	61	1.51E-25
GO:0016337, cell-cell adhesion	33	8.98E-17
GO:0007156, homophilic cell adhesion	25	4.41E-16
GO:0009605, response to external stimulus	31	5.78E-06
GO:0009611, response to wounding	25	6.36E-06
GO:0048518, positive regulation of biological process	38	3.50E-04
GO:0032501, multicellular organismal process	85	4.84E-04
GO:0032502, developmental process	77	4.97E-04
GO:0006954, inflammatory response	18	5.46E-04
GO:0048731, system development	50	5.76E-04
GO:0048856, anatomical structure development	57	6.46E-04
GO:0048513, organ development	40	9.45E-04
GO:0006950, response to stress	35	1.76E-03
GO:0006952, defense response	24	1.79E-03
GO:0007275, multicellular organismal development	59	1.80E-03
GO:0008283, cell proliferation	29	1.90E-03
GO:0007167, enzyme linked receptor protein signaling pathway	16	3.11E-03
GO:0001944, vasculature development	13	4.01E-03
GO:0048522, positive regulation of cellular process	32	4.99E-03
GO:0048523, negative regulation of cellular process	34	9.29E-03
GO:0009887, organ morphogenesis	18	1.50E-02
GO:0001568, blood vessel development	12	1.51E-02
GO:0006959, humoral immune response	8	1.52E-02
GO:0048519, negative regulation of biological process	34	1.77E-02
Enriched GO biological process terms in genes positively correlated with fatty acid synthesis pathways genes		
GO:0008610, lipid biosynthetic process	7	1.83E-03
GO:0000278, mitotic cell cycle	7	2.01E-03
GO:0007049, cell cycle	9	2.03E-03
GO:0007051, spindle organization and biogenesis	4	2.22E-03
GO:0000226, microtubule cytoskeleton organization and biogenesis	5	2.35E-03
GO:0000087, M phase of mitotic cell cycle	6	2.47E-03
GO:0006695, cholesterol biosynthetic process	4	2.52E-03
GO:0007067, mitosis	6	2.71E-03
GO:0051301, cell division	6	3.06E-03
GO:0016126, sterol biosynthetic process	4	3.46E-03
GO:0000279, M phase	6	5.39E-03
GO:0022403, cell cycle phase	6	1.39E-02
GO:0044255, cellular lipid metabolic process	7	1.48E-02

**Table IV:** Enriched GO biological process terms with the NFY-correlated genes.

Term	Count	Benjamini
GO:0006259, DNA metabolic process	16	1.49E-04
GO:0006139, nucleobase, nucleoside, nucleotide and nucleic acid metabolic process	31	4.00E-04
GO:0006974, response to DNA damage stimulus	10	1.05E-03
GO:0043170, macromolecule metabolic process	40	1.12E-03
GO:0006260, DNA replication	9	1.24E-03
GO:0006281, DNA repair	9	1.36E-03
GO:0009719, response to endogenous stimulus	10	2.74E-03
GO:0044238, primary metabolic process	41	1.07E-02
GO:0044237, cellular metabolic process	41	1.11E-02
GO:0006261, DNA-dependent DNA replication	6	1.20E-02
GO:0043283, biopolymer metabolic process	32	1.23E-02
GO:0016071, mRNA metabolic process	8	2.00E-02

**Table V:** List of genes in subnets 1 and 2 that were positively associated with the NFY-correlated genes.

Symbol	Name
Genes in subnet 1	
ASPM	ASP (abnormal spindle) homolog, microcephaly associated (*Drosophila*)
CPSF6	Cleavage and polyadenylation specific factor 6, 68 kDa
FBXO5	F-box protein 5
HAT1	Histone acetyltransferase 1
MCM2	MCM2 minichromosome maintenance deficient 2, mitotin (*S. cerevisiae*)
MDC1	Mediator of DNA damage checkpoint 1
MSH2	MutS homolog 2, colon cancer, nonpolyposis type 1 (*E. coli*)
MSH6	MutS homolog 6 (*E. coli*)
NUP153	Nucleoporin 153 kDa
ORC2L	Origin recognition complex, subunit 2-like (yeast)
RFC3	Replication factor c (activator 1) 3, 38 kDa
SS18	Synovial sarcoma translocation, chromosome 18
TDG	Thymine-DNA glycosylase
TRIM37	Tripartite motif-containing 37
UNG	Uracil-DNA glycosylase
Genes in subnet 2	
CBX1	Chromobox homolog 1 (hp1 β homolog *Drosophila*)
DEK	Dek oncogene (DNA binding)
LSM2	LSM2 homolog, U6 small nuclear RNA associated (*S. cerevisiae*)
NASP	Nuclear autoantigenic sperm protein (histone-binding)
PAIP1	Poly(a) binding protein interacting protein 1
RIT1	Ras-like without caax 1
SNRPE	Small nuclear ribonucleoprotein polypeptide e
TUBB	Tubulin, β
UPF2	UPF2 regulator of nonsense transcripts homolog (yeast)
